# Relationship between dietary pattern and depressive symptoms: an international multicohort study

**DOI:** 10.1186/s12966-023-01461-x

**Published:** 2023-06-20

**Authors:** Hanzhang Wu, Yeqing Gu, Ge Meng, Qing Zhang, Li Liu, Hongmei Wu, Shunming Zhang, Tingjing Zhang, Xuena Wang, Juanjuan Zhang, Shaomei Sun, Xing Wang, Ming Zhou, Qiyu Jia, Kun Song, Hong Chang, Tao Huang, Kaijun Niu

**Affiliations:** 1 School of Public Health, Tianjin University of Traditional Chinese Medicine, 10 Poyanghu Road, West Area, Tuanbo New Town, Tianjin, Jinghai District 301617 China; 2 School of Integrative Medicine, Tianjin University of Traditional Chinese Medicine, Tianjin, China; 3 Nutritional Epidemiology Institute and School of Public Health, Tianjin Medical University, Tianjin, China; 4 Institute of Radiation Medicine, Chinese Academy of Medical Sciences & Peking Union Medical College, Tianjin, China; 5 Department of Toxicology and Health Inspection and Quarantine, School of Public Health, Tianjin Medical University General Hospital, Tianjin, China; 6 Health Management Centre, Tianjin Medical University General Hospital, Tianjin, China; 7 Department of Epidemiology and Biostatistics, School of Public Health, Peking University Health Science Center, Beijing, China; 8 Tianjin Key Laboratory of Environment, Nutrition and Public Health, Tianjin, China; 9 Center for International Collaborative Research on Environment, Nutrition and Public Health, Tianjin, China

**Keywords:** Dietary pattern, Depressive symptoms, Cohort studies, Adults

## Abstract

**Background:**

Several previous studies have shown that dietary patterns are associated with the incidence of depressive symptoms. However, the results have been inconsistent. This study aimed to prospectively investigate the association between dietary patterns and the risk of depressive symptoms in two large cohort studies.

**Methods:**

The Tianjin Chronic Low-grade Systemic Inflammation and Health (TCLSIH) cohort study included a total of 7,094 participants living in Tianjin, China from 2013 to 2019, and the UK Biobank cohort study includes 96,810 participants who were recruited from 22 assessment centers across the UK taken between 2006 and 2010. All participants were free of a history of cardiovascular disease (CVD), cancer, and depressive symptoms at baseline. Dietary patterns at baseline were identified with factor analysis based on responses to a validated food frequency questionnaire in TCLSIH or Oxford WebQ in UK Biobank. Depressive symptoms were evaluated using the Chinese version of the Zung Self-Rating Depression Scale (SDS) in TCLSIH or hospital inpatient records in UK Biobank. Cox proportional hazards regression models were used to estimate the association between dietary patterns and depressive symptoms.

**Results:**

A total of 989, and 1,303 participants developed depressive symptoms during 17,410 and 709,931 person-years of follow-up. After adjusting for several potential confounders, the multivariable HRs (95% CIs) of the depressive symptoms were 0.71 (0.57, 0.88) for traditional Chinese dietary pattern, 1.29 (1.07, 1.55) for processed animal offal included animal food dietary pattern, and 1.22 (1.02, 1.46) for sugar rich dietary pattern in TCLSIH (all Q4 vs Q1). In the UK Biobank, the HRs (95% CIs) of depressive symptoms were 1.39 (1.16, 1.68) for processed food dietary pattern (Q4 vs Q1), 0.90 (0.77, 1.00) for healthy dietary pattern (Q3 vs Q1), and 0.89 (0.75, 1.05) for meat dietary pattern (Q4 vs Q1) in the final adjusted model.

**Conclusion:**

Dietary patterns rich in processed foods were associated with a higher risk of depressive symptoms, and following a traditional Chinese dietary pattern or healthy dietary pattern was associated with a lower risk of depressive symptoms, whereas meat dietary pattern was not associated.

**Supplementary Information:**

The online version contains supplementary material available at 10.1186/s12966-023-01461-x.

## Introduction

Depression is a common but serious mental health disorder, with an estimated 3.8% of the population affected [1], which has become a major factor in the decline in quality of life and is also associated with premature death [2]. It is estimated that the current treatment can only reduce the disease burden of patients with depressive symptoms by one-third [3]. The public health burden associated with depressive symptoms may be reduced through the identification of modifiable lifestyle factors (e.g., dietary factors) for early prevention.

Previous studies have demonstrated that some single foods or nutrients may affect the incidence of depressive symptoms [4–6]. However, people do not eat isolated nutrients or food. In addition, the food they eat may consist of a variety of kinds that provide complex combinations of nutrition for the human body. Due to the interaction and synergy among nutrients, single food or nutrient studies can’t well reflect the overall effect of food combinations. Therefore, the present study considered the whole diet rather than individual nutrients or foods to examine the complex association between dietary factors and the risk of depressive symptoms [7].

To date, results from several previous cross-sectional studies [8–10] and case–control studies [11, 12] in adults investigating the association between dietary patterns and depressive symptoms are mixed; however, these studies have methodological limitations, such as the cross-sectional design. In addition, few prospective studies have shown that healthy dietary pattern was inversely associated with the risk of depressive symptoms, whereas the Western dietary pattern was positively associated with the risk of depressive symptoms [13–15]. In contrast, a prospective study of US females did not find an association between the prudent or Western pattern and depressive symptoms risk [16]. Of note, these limited prospective studies were conducted in Western countries, so their results may not be generalizable to Chinese populations with different eating habits and lifestyles. Furthermore, dietary habits and incidence of depressive symptoms within distinct races were different [17, 18]. Therefore, we examined the associations of dietary patterns with the incidence of depressive symptoms in two large cohort studies: the Tianjin Chronic Low-grade Systemic Inflammation and Health (TCLSIH) and the UK Biobank.

## Methods

### Study population

The TCLSIH cohort study is an ongoing dynamic prospective cohort that began in 2007. The enrolled participants included men and women aged 18–90 years living in Tianjin, China, for at least 5 years. In this study, the participants were randomly recruited while having their annual health examinations at the Tianjin Medical University General Hospital-Health Management Center, the largest and most comprehensive physical examination center in Tianjin. The exclusion criteria were disability, severe mental illness, severe cognitive impairment, hearing impairment, and pregnancy. The overall response rate was 97.3%. Participants in this study covered nearly all occupations as well as retired residents. Examinations were usually performed in the same month every year, and participants were asked to complete a structured self-administered health status questionnaire according to their actual situation while having health examinations. We can obtain information about their socioeconomic status, mental health, physical activity, food intake, in addition to other related information through questionnaires. The Institutional Review Board of Tianjin Medical University has approved this study’s protocols and procedures (reference number: TMUh-MEC 201,430). Besides, all the participants have written informed consent.

**Fig. 1:**
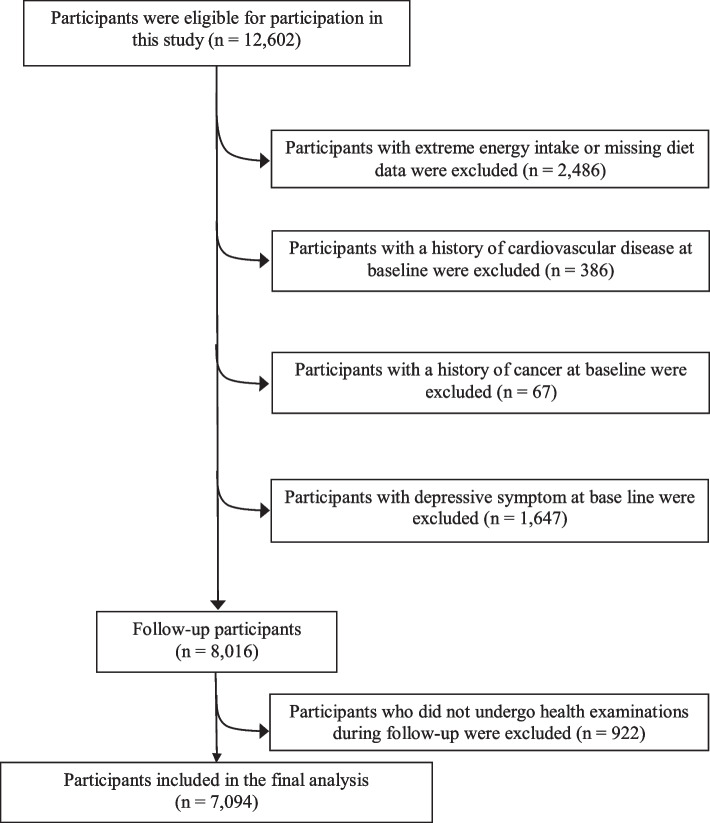
Selection of study participants in the TCLSIH Cohort

The eligible participants were followed from May 2013 to December 2019. During the study period, 11,136 adults who had received at least one health examination and returned the questionnaire. We excluded those with a history of cardiovascular disease (CVD) (*n* = 486), cancer (*n* = 75), had missing data or extreme total energy intake [< 542 (below the 2.5 percentile) or > 5,721 (over the 97.5 percentiles) kcal/day for men, and < 660 (below the 2.5 percentile) or > 4,947 (over the 97.5 percentiles) kcal/day for women; *n* = 913], or had depressive symptoms at baseline (*n* = 1,646). Besides, the participants who did not finish follow-up health examinations were excluded (*n* = 922, retention rate: 88.5%). Finally, 7,094 participants were included in the cohort analysis (Fig. 1).

The UK Biobank is a prospective population-based cohort study that recruited half a million men and women (39–72 years old) from the general population between 2006 and 2010 [19]. The UK Biobank protocol is available online (http://www.ukbiobank.ac.uk/wp-content/uploads/2011/11/UK-Biobank-Protocol.pdf). Participants had to be registered with a general practitioner and live within 25 miles of an assessment center (England, Wales, and Scotland) to take part. Participants provided extensive information through questionnaires, interviews, health records, physical measurements, and samples of blood and urine. The UK Biobank Study was approved by the North West Multi-centre Research Ethics Committee (REC reference for UK Biobank 11/NW/0274). At the touchscreen, all participants gave informed consent using a signature-capture device.

**Fig. 2:**
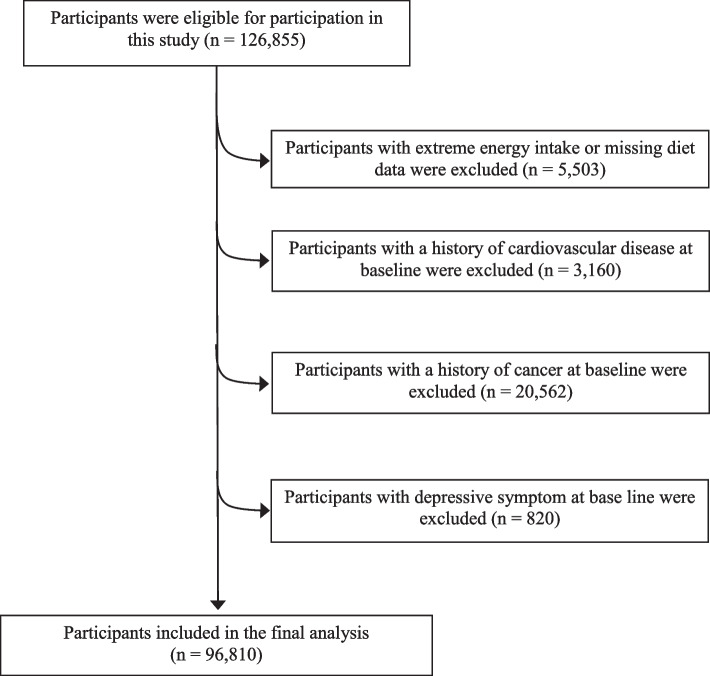
Selection of study participants in the UK Biobank

In the UK Biobank, participants who have completed at least two web-based 24-h dietary assessments were assessed (*n* = 126,855). We excluded those with a history of CVD (*n* = 3,160), cancer (*n* = 20,562), and depressive symptoms (*n* = 820) before the first diet recall. We also excluded participants who had unreliable total energy estimated intake data [< 800 or > 4,200 kcal/day for men, and < 600 or > 3,500 kcal/day for women; *n* = 5,503], a total of 96,810 participants were finally included in our study (Fig. 2).

### Assessment of depressive symptoms

In the TCLSIH cohort study, we used the Chinese version of the Zung Self-Rating Depression Scale (SDS) to evaluate depressive symptoms [20]. This scale was designed by Zung in 1965. The SDS has 20 items, each item has 1 to 4 points. The total score ranges from 20 to 80, and a higher score indicates depressive symptoms were serious. We set the cutoff score to 45 in our present study, a score higher than that can be considered depressive symptoms. We conducted a screening test to calculate the sensitivity and specificity of the SDS in the study population (120 patients with clinically diagnosed depressive symptoms and 120 healthy controls). When the SDS score of 45 was used as a cutoff and the Diagnostic and Statistical Manual of Mental Disorders (fourth edition) was used as the criteria for depressive symptoms, the sensitivity and specificity were 83.6% and 96.4%, respectively [12].

In the UK biobank, diagnoses of depressive symptoms were recorded using the International Classification of Diseases (version 10; code ICD-10) coding system. Participants with depressive symptoms were identified as having a primary/secondary diagnosis (hospital records) using ICD-10 codes.

### Dietary assessment

In the TCLSIH cohort study, we used the food frequency questionnaire (FFQ) which included 100 food items with specified serving sizes to assess the dietary intake since May 2013. Each food in this FFQ has seven frequencies (ranging from “almost never” to “two or more times/day”) and eight frequencies for beverages consumed (ranging from “almost never” to “four or more times/day”). All participants were asked how often they had consumed each food by choosing from predefined frequency categories on average last month. The reproducibility and validity of the questionnaire were assessed in a random sample of participants from our cohort using data from repeated measurements of the FFQ approximately 3 months apart and 4-day weighed diet records (WDRs). The participants for the validation were randomly selected from different subgroups (age: 20–30, 30–40, 40–50, 50–60, 60–70, and > 70 years) of the TCLSIH cohort study participants, and at least 10 men and 10 women were included in each of these subgroups. The Spearman correlation coefficients between the two FFQs were 0.68 for energy intake, and 0.62–0.79 for food items (e.g., fruits, vegetables, and beverages). The Spearman correlation coefficients between the gold standard (WDRs) and FFQs were 0.49 for total energy intake, 0.39–0.72 for energy-adjusted nutrients (e.g., vitamin C, vitamin E, polyunsaturated fatty acid, saturated fatty acids, carbohydrates). The total energy and nutrient intake (kcal/day) was calculated by using an ad hoc computer program developed to analyze the FFQ and the nutrient database derive from the Chinese Food Composition Tables [21]. We categorized the food items and beverages from the FFQ into 30 predefined food groups,

In the UK Biobank, dietary information was collected using the Oxford WebQ, a web-based self-administered 24 h dietary assessment questionnaire developed for using in large population studies. UK Biobank participants were invited up to five times to complete the Oxford WebQ dietary assessment, and we calculated mean values from the available data. The Oxford WebQ dietary assessment asks about the consumption of over 200 common food and beverage items in the previous 24 h [22]. We created new variables for the weight (gram) from the 24-h dietary assessments to estimate the daily intake of each food or drink. The top frequency category was open-ended so we coded 3 +  = 3, 4 +  = 4, 5 +  = 5, 6 +  = 6 and less than one was coded as 0.5. Weight was generated by multiplying the frequency of consumption by the serving size in grams. Finally, we classified foods and beverages reported in the Oxford WebQ into 14 main food categories (12 food and 2 beverage categories, Spreads, sauces, and cooking oil were excluded) based on a previous study [23].

### Identification of dietary patterns

Due to the differences in food selection within distinct races, we categorized the food items and beverages from the TCLSIH cohort into 30 predefined food groups and 14 food groups in the UK Biobank cohort based on nutrient and culinary similarities [23]. Dietary patterns were identified using factor analysis (principal component analysis) based on the food groups in these two cohorts. The factors were rotated by an orthogonal transformation (varimax rotation function in SAS software) to achieve a simple structure, allowing greater interpretability. After combining eigenvalues criteria (> 1.5), Scree plot test, greater interpretability of factors, and percentage of variance explained by the factors, factors were named descriptively based on food items with high factor loading. In the TCLSIH cohort study, three factors were named traditional Chinese dietary pattern, processed animal offal included animal food dietary pattern, and sugar rich dietary pattern. And in the UK Biobank study, three factors were named as healthy dietary pattern, processed food dietary pattern, and meat dietary pattern. Factor loadings represented the correlation coefficient between food groups and specific dietary patterns, where positive loadings represented positive correlation and negative represented inverse correlation. The dietary pattern score for each pattern was calculated by summing observed intakes of food groups weighted by their factor loadings. A higher score suggested greater adherence to a certain dietary pattern. Dietary pattern scores were standardized to have a mean of 0 and a standard deviation of 1. A higher dietary pattern score means greater conformity to this dietary pattern, contrarily unrelated.

### Assessment of baseline covariates

In TCLSIH, sociodemographic characteristics (age, sex, education level, occupation, monthly household income, living alone, and the frequency of visiting friends), lifestyle, family and personal disease histories, and current medicines were obtained from a detailed and structured health-related questionnaire. All participants received a standardized physical examination, to obtain the height and weight data. Body mass index (BMI, kg/m^2^) was calculated using weight divided by height square. Physical activity (PA) in recent weeks was assessed by using the short form of the International Physical Activity Questionnaire [24]. Total PA levels were calculated according to the following formula: metabolic equivalent (MET) × hour/week.

All the participants were asked to fast overnight before collecting blood samples. Total cholesterol (TC), total triglycerides (TG), low‐density lipoprotein cholesterol (LDL-C), and high-density lipoprotein cholesterol (HDL-C) were measured using an automatic biochemistry analyzer (Roche Cobas 8000 modular analyzer, Mannheim, Germany). Fasting blood glucose (FBG) was measured by enzymatic methods; glycated hemoglobin (HbA1c) was measured by high-performance liquid chromatographic method (HLC-723 G8; Tosoh, Tokyo, Japan); 2-h serum glucose was measured at 2 h after administration during the oral glucose tolerance test. Diabetes was defined as FBG level ≥ 7.0 mmol/L, and/or 2-h serum glucose ≥ 11.1 mmol/L, and/or HbA1c ≥ 48 mmol/mol (6.5%), and/or taking hypoglycemic drugs [25]. Blood pressure (BP) was measured on the upper left arm with an automatic device (TM-2655P, A&D Company, Ltd., Tokyo, Japan). The participants’ BP was measured twice and calculated the mean of the two data. Hypertension was defined as systolic/diastolic blood pressure ≥ 140/90 mm Hg or having a history of hypertension or use of antihypertension medications [26]. Hyperlipidemia was defined as TC ≥ 5.17 mmol/L, and/or TG ≥ 1.7 mmol/L, and/or LDL ≥ 3.37 mmol/L, and/or taking lipid-lowering drugs.

In the UK Biobank, the baseline questionnaire was used to assess several possible confounding variables including age, sex, smoking status, alcohol drinking status, whether they are living alone, and the frequency of visiting friends. We also collect information about comorbidities and family history of the disease. Townsend deprivation index was derived from the postcode of residence using aggregated data on unemployment, car and homeownership, and household overcrowding [27].

### Statistical analysis

The normality of the continuous variables was examined by the Kolmogorov–Smirnov test (n > 2000). Baseline characteristics of the study participants by depressive symptoms status were presented as geometric mean and 95% confidence intervals (CIs) for continuous variables and percentages for categorical variables. The differences between participants with and without incident depressive symptoms were examined by using the *T* test for continuous variables, and the χ^2^ test for proportional variables.

Person-years of follow-up for each participant in TCLSIH were calculated from the date when the participants first completed their FFQ survey and ended at the date of the first diagnosis of depressive symptoms, the end of follow-up (31 December 2019), or loss to follow-up, whichever was earliest. Follow-up time in the UK Biobank was calculated from the date of first completed the Oxford WebQ dietary assessment until the date of incident depressive symptoms, death, loss to follow-up, or end of study (January 31, 2018). Cox proportional hazards regression models were used to evaluate the relationship between the dietary pattern and the risk of depressive symptoms. The proportional hazards assumption was tested by Schoenfeld residuals and no violation of this assumption was found in our analyses. In further analysis, the dietary pattern score was set as independent variable in quartile categories and depressive symptoms were set as the dependent variable. Three models were fitted for the outcomes. Model 1 was adjusted for age, sex, and BMI. Model 2 was additionally adjusted for smoking status, alcohol drinking status, education levels, household income (only in the TCLSIH cohort), employment status (only in the TCLSIH cohort), Townsend deprivation index (only in the UK Biobank), total energy intake, physical activity, frequency of visiting friends, living alone based on model 1. Model 3 was additionally adjusted for family history of diseases (hypertension, CVD, hyperlipidemia (only in TCLSIH cohort), and diabetes), and individual history of the disease (hypertension, hyperlipidemia (only in TCLSIH cohort), and diabetes). We also did sensitivity analyses by including a total of 163,476 participants who completed ≥ 1 web-based 24-h dietary assessment and were without CVD, cancer, and depressive symptoms at baseline in the UK Biobank cohort.

Hazard ratios with their corresponding 95% CIs were calculated and two-sided *P* < 0.05 was considered as statistically significant. The linear trend test was performed by assigning medians to each quintile as a continuous variable in the model. The median value for each quartile was used to test for trends across quartiles. The likelihood ratio test was used to assess the significance of the variables. The statistical analyses were performed by SAS version 9.3 (SAS Institute Inc., Gary, NC, USA).

## Results

Tables 1 and 2 show the top 15 (TCLSIH) and all UK Biobank food items and factor loads for each dietary pattern extracted by factor analysis. In the TCLSIH cohort study, these three patterns explained 31.3% of the variance in dietary intake. The first pattern was characterized by a high intake of vegetables and grains, therefore, was identified as a traditional Chinese dietary pattern. The second pattern consisted of a high intake of organ meat, red meat, processed meat, and preserved food named processed animal offal included animal food dietary pattern. The third pattern had high factor loadings for dim sum, fruits, desserts, and sweetened beverages, and was named the sugar rich dietary pattern. In the UK Biobank study, the three dietary patterns were named healthy dietary pattern, processed food dietary pattern, and meat dietary pattern according to the factor loadings of the food group. These three patterns explained 31.9% of the variance in dietary intake.

**Table 1:** Factor loadings for food items derived from factor analysis (principal component analysis) in the TCLSIH Cohort Study ^a^

Traditional Chinese dietary pattern		Processed animal offal included animal food dietary pattern		Sugar rich dietary pattern	
**Food items**	**Factor loadings**	**Food items**	**Factor loadings**	**Food items**	**Factor loadings**
Solanaceous vegetables	0.72	Organ meats	0.68	Dim sum (Cakes, cookies, and biscuits)	0.56
Dark green and leafy vegetables	0.67	Red meats	0.55	Fruits (all kinds except for persimmon, strawberry, and kiwi fruit)	0.54
Starchy vegetables	0.65	Animal blood	0.54	Fruits (persimmon, strawberry, kiwi fruit)	0.53
Mushroom and fungi	0.61	Steamed stuffed bun, dumpling, and wonton	0.52	Sweets and desserts	0.49
Legumes and soy products	0.58	Preserved food	0.52	Soda and sweetened beverages	0.43
Whole grains	0.48	Noodles	0.49	Bread	0.37
Refined grains	0.48	Sausage	0.48	Nuts	0.34
Allium vegetables	0.47	Poultry	0.46	Coffee	0.33
Chicken egg	0.46	Fish and Seafood	0.42	Salted food	0.33
Fruits (all kinds except for persimmon, strawberry, and kiwi fruit)	0.42	Salted food	0.36	Preserved food	0.28
Nuts	0.37	Soda and sweetened beverages	0.31	Starchy vegetables	0.28
Poultry	0.33	Alcohol consumption	0.29	Allium vegetables	0.27
Fish and Seafood	0.33	Allium vegetables	0.24	Dairy products	0.26
Red meats	0.31	Sweets and desserts	0.20	Animal blood	0.24
Dairy products	0.27	Dairy products	0.15	Fish and Seafood	0.22
**Variance explained (%)**	12.8	**Variance explained (%)**	10.0	**Variance explained (%)**	8.5

^a^For simplicity, only the top fifteen food items of each pattern are shown

**Table 2:** Factor loadings for food items derived from factor analysis (principal component analysis) in the UK Biobank Cohort Study ^a^

Healthy dietary pattern		Processed food dietary pattern		Meat dietary pattern	
**Food groups**	**Factor loadings**	**Food groups**	**Factor loadings**	**Food groups**	**Factor loadings**
Vegetables & potatoes	0.71	Fat & spreads	0.68	Meat & meat products	0.78
Fruits	0.70	Cereals & cereal products	0.60	Alcoholic beverages	0.50
Dairy & dairy-free products	0.41	Sugar, preserves, cakes & confectionery, snacks	0.57	Egg & egg dishes	0.26
Nuts & seeds	0.34	Non-alcoholic beverages	0.51	Vegetables & potatoes	0.15
Non-alcoholic beverages	0.31	Meat & meat products	0.20	Non-alcoholic beverages	0.09
Fish & fish dishes	0.31	Mixed-dishes	0.13	Sugar, preserves, cakes & confectionery, snacks	0.06
Egg & egg dishes	0.25	Dairy & dairy-free products	0.13	Fat & spreads	0.05
Meat substitutes	0.08	Meat substitutes	0.09	Nuts & seeds	0.02
Cereals & cereal products	0.07	Vegetables & potatoes	0.05	Fish & fish dishes	-0.11
Meat & meat products	0.02	Fruits	0.04	Mixed-dishes	-0.13
Mixed-dishes	0.02	Egg & egg dishes	0.03	Dairy & dairy-free products	-0.17
Sugar, preserves, cakes & confectionery, snacks	-0.09	Nuts & seeds	-0.04	Fruits	-0.19
Alcoholic beverages	-0.10	Alcoholic beverages	-0.05	Cereals & cereal products	-0.21
Fat & spreads	-0.15	Fish & fish dishes	-0.15	Meat substitutes	-0.53
**Variance explained (%)**	11.3	**Variance explained (%)**	10.8	**Variance explained (%)**	9.8

^a^participants who completed at least two web-based 24-h dietary were assessment in the UK Biobank cohort

The baseline characteristics of the study participants from the TCLSIH cohort and UK Biobank are shown in Table 3. In the TCLSIH cohort study, 7,094 participants were enrolled, and within 17,410 person-years of follow-up, 989 participants developed depressive symptoms. In UK Biobank, 96,810 participants were enrolled, and within 709,931 person-years of follow-up, 1303 participants developed depressive symptoms.

**Table 3:** Baseline participant characteristics by status of depressive symptoms ^a^

Characteristics	All	Depressive symptoms status		*P* value ^b^
		No	Yes	
**The TCLSIH**				
No. of participants	7,094	6,105	989	-
Age (years)	38.1 (37.9, 38.3) ^c^	38.0 (37.8, 38.3)	38.4 (37.8, 39.0)	0.21
Sex (male, %)	56.8	57.0	56.0	0.58
BMI (kg/m^2^)	24.1 (24.0, 24.2)	24.2 (24.1, 24.2)	24.0 (23.8, 24.2)	0.18
TC (mmol/L)	4.60 (4.60, 4.70)	4.60 (4.60, 4.70)	4.60 (4.60, 4.70)	0.41
TG (mmol/L)	1.13 (1.12, 1.15)	1.13 (1.12, 1.15)	1.14 (1.10, 1.18)	0.87
LDL-C (mmol/L)	2.64 (2.62, 2.66)	2.64 (2.62, 2.66)	2.64 (2.59, 2.69)	0.96
HDL-C (mmol/L)	1.33 (1.32, 1.34)	1.33 (1.32, 1.34)	1.31 (1.29, 1.34)	0.19
FBG (mmol/L)	5.06 (5.04, 5.07)	5.06 (5.04, 5.08)	5.04 (5.00, 5.08)	0.43
SBP (mmHg)	119.5 (119.2, 119.9)	119.5 (119.2, 119.9)	119.3 (118.3, 120.2)	0.60
DBP (mmHg)	75.6 (75.4, 75.9)	75.7 (75.4, 76.0)	75.4 (74.7, 76.1)	0.38
PA (MET × hour/week)	10.8 (10.5, 11.1)	11.0 (10.7, 11.4)	9.80 (9.00, 10.6)	< 0.01
Total energy intake (kcal/day)	2283.9 (2263.6, 2304.3)	2284.6 (2262.9, 2306.4)	2279.8 (2223.6, 2337.5)	0.88
"Vegetable rich" dietary pattern score	0.00 (-0.02, 0.02)	0.01 (-0.01, 0.04)	-0.08 (-0.14, -0.02)	< 0.01
"Sugar rich" dietary pattern score	0.00 (-0.02, 0.02)	-0.01 (-0.03, 0.02)	0.04 (-0.03, 0.11)	0.20
"Animal foods" dietary pattern score	0.00 (-0.02, 0.02)	-0.02 (-0.05, 0.00)	0.14 (0.07, 0.21)	< 0.0001
Smoking status (%)				0.01
Current smoker	17.9	17.3	21.3	
Ex-smoker	5.21	5.29	4.70	
Non-smoker	76.9	77.4	74.0	
Drinking status (%)				0.52
Everyday	3.10	2.98	3.80	
Sometime	61.3	61.2	61.6	
Ex-drinker	8.98	9.06	8.52	
Non-drinker	26.7	26.7	26.1	
Married (%)	85.2	85.2	85.1	0.93
Living alone (%)	7.45	7.28	8.47	0.19
Education level (college or higher, %)	80.9	82.1	73.7	< 0.0001
Occupation (%)				0.48
Managers	51.8	52.0	50.3	
Professionals	18.5	18.5	18.3	
Other	29.8	29.5	31.4	
Visiting friends (%)	55.6	56.4	50.7	< 0.001
Household income (≥ 10,000 Yuan, %)	46.8	48.4	36.8	< 0.0001
Individual history of disease (%)				
Hypertension	21.2	21.2	21.2	0.99
Hyperlipidemia	43.8	43.5	41.0	0.13
Diabetes	4.75	4.83	4.25	0.42
Family history of disease (%)				
CVD	33.7	33.6	34.4	0.62
Hypertension	55.8	55.5	57.4	0.28
Hyperlipidemia	0.49	0.47	0.61	0.47
Diabetes	29.7	29.5	31.4	0.23
**The UK Biobank**				
No. of participants	96,810	95,507	1,303	-
Age (years)	54.8 (54.8, 54.9)	54.8 (54.8, 54.9)	54.4 (54.0, 54.8)	0.03
Sex (male, %)	44.1	44.2	31.9	< 0.0001
BMI (kg/m^2^)	26.3 (26.2, 26.3)	26.2 (26.2, 26.3)	27.9 (27.6, 28.2)	< 0.0001
SBP (mmHg)	132.9 (132.8, 133.0)	132.9 (132.8, 133.1)	131.1 (130.0, 132.3)	< 0.01
DBP (mmHg)	79.8 (79.7, 79.9)	79.8 (79.7, 79.9)	79.7 (79.0, 80.3)	0.65
Townson depretive index	-1.62 (-1.64, -1.60)	-1.63 (-1.64, -1.61)	-1.11 (-1.28, -0.94)	< 0.0001
PA (MET × hour/week)	26.0 (25.8, 26.2)	26.1 (25.9, 26.2)	22.0 (20.5, 23.6)	< 0.0001
Total energy intake (kcal/day)	2039.2 (2035.8, 2042.5)	2039.6 (2036.2, 2042.9)	2009.8 (1979.9, 2040.1)	0.06
"Healthy" dietary pattern score	0.00 (-0.01, 0.01)	0.00 (-0.01, 0.01)	0.05 (-0.01, 0.11)	0.13
"Processed food" dietary pattern score	0.00 (-0.01, 0.01)	0.00 (-0.01, 0.00)	0.10 (0.04, 0.16)	< 0.001
"Meet" dietary pattern score	0.00 (-0.01, 0.01)	0.00 (-0.01, 0.01)	-0.04 (-0.10, 0.02)	0.17
Smoking status (%)				< 0.0001
Current smoker	6.87	6.79	12.2	
Ex-smoker	34.6	34.5	37.2	
Non-smoker	58.6	58.7	50.6	
Drinking status (%)				< 0.0001
Current drinker	94.5	94.6	89.3	
Ex-drinker	2.70	2.64	6.68	
Non-drinker	2.79	2.77	3.99	
Living alone (%)	17.6	17.5	23.9	< 0.0001
Education level (college or higher, %)	47.6	47.7	40.2	< 0.0001
Visiting friends (≥ once a week, %)	74.9	74.9	76.1	0.34
Individual history of disease (%)				
Hypertension	21.1	21.0	28.1	< 0.0001
Diabetes	2.84	2.80	5.53	< 0.0001
Family history of disease (%)				
CVD	54.0	53.9	58.9	< 0.001
Hypertension	44.2	44.2	46.2	0.14
Diabetes	16.9	16.9	18.7	0.07

^a^Continuous variables are expressed as means (± standard deviation, SD) and categorical variables are expressed as percentages. BMI, body mass index; CVD, cardiovascular disease; DBP, diastolic blood pressure; FBG, fasting blood glucose; HDL-C, high-density lipoprotein cholesterol; LDL-C, low-density lipoprotein cholesterol; MET, metabolic equivalent; PA, physical activity; SBP, systolic blood pressure; TC, total cholesterol; TG, triglycerides

^b^*T* test or χ^2^ test

^c^Geometric mean (95% confidence interval) (all such values)

In brief, in the TCLSIH cohort, compared with subjects who did not develop depressive symptoms, those participants with incident depressive symptoms tended to have a lower proportion of education level, visiting friends, and household income (all *P* values < 0.05). In the UK Biobank, compared with subjects who did not develop depressive symptoms, those participants with incident depressive symptoms tended to have a higher BMI, Townson deprivation index, and a higher proportion of females, living alone, hypertension, diabetes, and a family history of CVD.

In Table 4, cox proportional hazards regression models show the association between dietary patterns and the risk of depressive symptoms. In the TCLSIH cohort study, after adjusting for demographic factors, lifestyle factors, disease history, and each other dietary pattern score, the final multivariate model shows that participants in the highest quartile of traditional Chinese dietary pattern score had a multivariable HR of 0.71 (95% CI: 0.57, 0.88; *P* for trend < 0.001), compared with those in the lowest quartile. For the same comparison, participants in the highest quartile of processed animal offal included animal food dietary pattern score had a multivariable HR of 1.29 (95% CI: 1.07, 1.55; *P* for trend < 0.01). Participants with a higher intake of the sugar rich dietary pattern had a multivariable HR of 1.22 (95% CI: 1.02, 1.46; *P* for trend = 0.03), for the same comparison. In the UK Biobank, the multivariable HRs (95% CIs) of depressive symptoms across the quartiles of the healthy dietary pattern were 1.00 (reference), 0.84 (0.72, 0.99), 0.90 (0.77, 1.00), and 0.97 (0.83, 1.14) (*P* for trend = 0.90) in the final model. For the same comparison, the multivariable HRs (95% CIs) of depressive symptoms across the quartiles of processed food dietary pattern were 1.00 (reference), 1.08 (0.92, 1.27), 1.34 (1.14, 1.58), and 1.39 (1.16, 1.68) (*P* for trend < 0.0001). Finally, the multivariable HRs (95% CIs) of depressive symptoms across the quartiles of processed food dietary pattern were 1.00 (reference), 0.99 (0.85, 1.15), 0.94 (0.81, 1.10), and 0.89 (0.75, 1.05) (*P* for trend = 0.13).

**Table 4:** Association between dietary patterns and risk of depressive symptoms in the TCLSIH and UK Biobank Cohort Study

	Quartiles of dietary pattern scores				*P* for trend ^a^
	Q1	Q2	Q3	Q4	
**The TCLSIH**					
**Traditional Chinese dietary pattern**					
No. of depressive symptoms	278	249	230	232	
Person years	4,255	4,375	4,392	4,389	
Incidence per 1000 person years	65.33	56.91	52.37	52.86	
Model 1	1.00 (reference)	0.86 (0.73, 1.02) ^b^	0.79 (0.66, 0.94)	0.79 (0.67, 0.94)	< 0.01
Model 2	1.00 (reference)	0.85 (0.72, 1.01)	0.78 (0.65, 0.94)	0.71 (0.57, 0.88)	< 0.001
Model 3	1.00 (reference)	0.85 (0.71, 1.01)	0.78 (0.65, 0.94)	0.71 (0.57, 0.88)	< 0.001
**Processed animal offal included animal food dietary pattern**					
No. of depressive symptoms	248	215	221	305	
Person years	4,365	4,391	4,360	4,295	
Incidence per 1000 person years	56.82	48.96	50.69	71.01	
Model 1	1.00 (reference)	0.88 (0.73, 1.06)	0.93 (0.77, 1.12)	1.32 (1.11, 1.56)	< 0.001
Model 2	1.00 (reference)	0.87 (0.72, 1.05)	0.93 (0.77, 1.12)	1.29 (1.07, 1.54)	< 0.01
Model 3	1.00 (reference)	0.87 (0.73, 1.05)	0.93 (0.77, 1.12)	1.29 (1.07, 1.55)	< 0.01
**Sugar rich dietary pattern**					
No. of depressive symptoms	232	241	250	266	
Person years	4,420	4,298	4,418	4,276	
Incidence per 1000 person years	52.49	56.07	56.59	62.21	
Model 1	1.00 (reference)	1.08 (0.90, 1.29)	1.08 (0.91, 1.30)	1.21 (1.01, 1.44)	0.04
Model 2	1.00 (reference)	1.09 (0.91, 1.31)	1.11 (0.93, 1.33)	1.22 (1.02, 1.46)	0.03
Model 3	1.00 (reference)	1.09 (0.91, 1.31)	1.11 (0.93, 1.33)	1.22 (1.02, 1.46)	0.03
**The UK Biobank**					
**Healthy dietary pattern**					
No. of depressive symptoms	349	289	310	355	
Person years	177,189	177,755	177,974	177,013	
Incidence per 1000 person years	1.97	1.63	1.74	2.01	
Model 1	1.00 (reference)	0.81 (0.69, 0.95)	0.85 (0.73, 1.00)	0.95 (0.81, 1.10)	0.65
Model 2	1.00 (reference)	0.85 (0.72, 0.99)	0.90 (0.77, 1.00)	0.98 (0.84, 1.15)	0.99
Model 3	1.00 (reference)	0.84 (0.72, 0.99)	0.90 (0.77, 1.00)	0.97 (0.83, 1.14)	0.90
**Processed food dietary pattern**					
No. of depressive symptoms	295	301	361	346	
Person years	177,848	177,906	177,636	176,541	
Incidence per 1000 person years	1.66	1.69	2.03	1.96	
Model 1	1.00 (reference)	1.04 (0.89, 1.22)	1.30 (1.11, 1.51)	1.35 (1.15, 1.58)	< 0.0001
Model 2	1.00 (reference)	1.09 (0.92, 1.28)	1.36 (1.16, 1.60)	1.43 (1.18, 1.72)	< 0.0001
Model 3	1.00 (reference)	1.08 (0.92, 1.27)	1.34 (1.14, 1.58)	1.39 (1.16, 1.68)	< 0.0001
**Meat dietary pattern**					
No. of depressive symptoms	336	335	324	308	
Person years	177,286	177,802	177,632	177,211	
Incidence per 1000 person years	1.90	1.88	1.82	1.74	
Model 1	1.00 (reference)	0.96 (0.83, 1.12)	0.92 (0.79, 1.08)	0.91 (0.78, 1.07)	0.21
Model 2	1.00 (reference)	0.99 (0.85, 1.15)	0.95 (0.81, 1.10)	0.90 (0.76, 1.05)	0.16
Model 3	1.00 (reference)	0.99 (0.85, 1.15)	0.94 (0.81, 1.10)	0.89 (0.75, 1.05)	0.13

^a^ Obtained by using multivariable Cox regression model

^b^ Hazard ratios (95% confidence interval) (all such values)

Model 1 was adjusted for age, sex, and body mass index

Model 2 was additionally adjusted for smoking status, alcohol drinking status, married (only in TCLSIH cohort), education level, occupation (only in TCLSIH cohort), visiting friends, living alone, household income (only in TCLSIH cohort), physical activity, total energy intake, Townson depressive index (only in UK Biobank)

Model 3 was additionally adjusted for family history of disease (including cardiovascular disease, hypertension, hyperlipidemia (only in TCLSIH cohort), and diabetes), hypertension, hyperlipidemia (only in TCLSIH cohort), diabetes

In sensitivity analyses, three dietary patterns remained similar when we include the participants who completed ≥ 1 web-based 24-h dietary assessment (Supplementary Table 1). Similar results were observed in the association between the three dietary patterns and depressive symptoms (Supplementary Table 2).

## Discussion

The objective of this study was to examine the association between dietary patterns from factor analysis and depressive symptoms in participants in the TCLSIH and UK Biobank. Results indicated that in both cohorts, greater adherence to healthy dietary patterns were associated with a lower risk of depressive symptoms, and more closely to the dietary patterns rich in processed foods was significantly associated with a higher risk of depressive symptoms. However, we did not observe significant associations between meat dietary pattern and depressive symptoms risk in the UK Biobank. This investigation has further confirmed the significant association between dietary patterns and the risk of depressive symptoms.

Our results reported a positive association between dietary patterns characterized by high intakes of processed food (i.e., sugar rich and processed animal offal included in the animal food dietary pattern in the TCLSIH and processed food dietary pattern in the UK Biobank) and depressive symptoms risk. Similarly, previous cross-sectional studies have shown that dietary patterns highly loaded with processed foods were associated with a higher risk of depressive symptoms [9, 28, 29]. Moreover, three prospective cohort studies indicated a positive association between processed food patterns (derived from principal component analysis) and depressive symptoms [13, 30, 31]. In contrast, one prospective study conducted in US middle-aged and older females found no significant associations between Western dietary patterns and the new onset of depression [16]. This inconsistency may be due to differences in the study populations, sex, race, and geographical area. Indeed, a recent meta-analysis reported that when the participants were Asian and (or) less than 50 years old, the associations between healthy or Western-style dietary pattern and depression were obvious [32]. There are several plausible mechanisms underlying this association. All these dietary patterns were characterized by a poor nutritional profile with higher energy density, total fat, saturated fat, added sugar and salt, and lower amounts of dietary fiber and vitamins [33]. The low levels of the brain-derived neurotrophic factor (BDNF) induced by higher sugar consumption, which has been discussed as facilitating neurogenesis and hippocampal atrophy in depressive symptoms [34, 35]. Moreover, a previous study demonstrated the Western diet with a higher intake of processed meat, sweets, desserts, French fries, and high-fat dairy products was associated with higher levels of C-reactive protein and interleukin-6 (markers of systemic inflammation) [36]. Proinflammatory cytokines alter the production, metabolism, and transport of neurotransmitters that synergistically affect mood, including dopamine, glutamate, and serotonin [37].

We found that the traditional Chinese dietary pattern and healthy dietary pattern were associated with decreased risk of depressive symptoms. Although the Chinese traditional diet has been westernized, including reducing the intake of vegetables and whole grains and increasing the intake of refined carbohydrates, added sugar, fat, and animal source foods [38], the persistence of Chinese culinary tradition was revealed from the dietary patterns identified in the TCLSIH cohort. A cross-sectional study of Japanese people found a healthy Japanese dietary pattern with high consumption of vegetables, fruit, soy products, and mushrooms was associated with a decreased incidence of depressive symptoms [8]. Furthermore, previous meta-analysis and systematic review of observational studies both indicated that healthy dietary pattern was associated with a decreased risk of depression [32, 39]. This association might be explained by dietary fiber in vegetables and whole grains. Dietary fiber can affect the diversity and composition of intestinal microflora structure [40], which can increase serotonin concentration and reduce the production of inflammatory cytokines [41, 42]. Meanwhile, the potential protective effect of the traditional Chinese dietary pattern could also come from the high content of antioxidants in vegetables such as vitamin C, vitamin E, and other carotenoids compounds, as some studies have shown higher antioxidant levels to be associated with a lower risk of depressive symptoms [43]. Interestingly, participants with the highest quartiles of healthy dietary pattern scores were not with a lower risk of depressive symptoms. That might be partly due to a higher intake of starchy vegetables which may not confer the same health benefits as other fruits and vegetables [44].

However, in the UK Biobank cohort, the higher consumption of meat dietary pattern was not associated with a high risk of depressive symptoms. Previous studies have some controversial reports on meat intake and depressive symptoms [30, 45]. The uncertain association may be due to the effect of white, red, and processed meat on depression may differ from each other [46]. In the present study, the various varieties of meat could only be regarded as a whole. That means the results of this study are subject to the combined effect of different varieties of meat. Furthermore, most of the participants in the UK Biobank cohort prefer to remove the fat from meat and remove the skin from poultry before cooking, which could also reduce the detrimental effect of meat intake on depression.

Strengths of this study include its large sample size of TCLSIH and UK Biobank participants, and a dietary assessment tool that allowed for detailed estimation of food and nutrient intakes as well as overall dietary patterns. Furthermore, these data allowed us to investigate the association between dietary patterns and depressive symptoms with diet collected before the onset of the disease, which can reduce reverse causal associations compared to cross-sectional studies. We also adjusted for a variety of covariables and conducted sensitivity analyses to confirm the robustness of derived dietary patterns and their association with depressive symptoms. However, a few limitations are notable in the present study. First, the statistical analysis used to derive dietary patterns involves several arbitrary decisions, including the construction of the food groups and the method of rotation. However, similar dietary patterns were identified when including individual food items instead of food groups in our previous study [47]. In addition, three dietary patterns remained similar when we included the participants who completed ≥ 1 WebQ dietary assessment in the UK Biobank study. Second, the measurement of depressive symptoms in the TCLSIH study depends on SDS rather than conducting diagnostic psychiatric interviews. However, the sensitivity and specificity of the SDS score for the assessment of depressive symptoms compared to the Diagnostic and Statistical Manual of Mental Disorders (fourth edition), were 83.6% and 96.4%, respectively. Third, dietary intake was assessed using the 100-item FFQ or WebQ dietary assessment which cannot include all the food items and could be subject to recall bias [48]. Therefore, the present dietary pattern may not reflect the real situation of the study population. Fourth, the study participants were mainly Chinese adults and British whites, and our findings may only apply to the population with similar demographics. Finally, even though many confounding factors have been taken into account, some potential residual factors are still unavoidable to confound the observed relationship.

## Conclusions

Based on two large China and UK cohorts, following a traditional Chinese dietary pattern or healthy dietary pattern was associated with a lower incidence of depressive symptoms, while a higher intake of a diet rich in processed food was associated with an increased risk of depressive symptoms. Furthermore, following a meat dietary pattern was not associated with depressive symptoms in the UK. These findings suggest that dietary modification may be a potential target for the prevention of depressive disorders. Further research is urgently required to confirm the causal association of dietary patterns with the risk of depressive symptoms, and they need to be confirmed in other populations and settings.

## Supplementary Information

**Additional file 1.** 

## Data Availability

TCLSIH data cannot be made publicly available because public availability would compromise participant privacy. For data access, researchers can contact the School of Public Health, Tianjin University of Traditional Chinese Medicine, Tianjin, China (E-mail address: nkj0809@gmail.com). UK Biobank data are available in a public, open-access repository. This research has been conducted using the UK Biobank Resource under Application Number 44430. The UK Biobank data are available on the application to the UK Biobank (www.ukbiobank.ac.uk/).

## Abbreviations

BDNF: Brain-derived neurotrophic factor
BMI: Body mass index
BP: Blood pressure
CVD: Cardiovascular disease
CI: Confidence interval
FBG: Fasting blood glucose
FFQ: Food frequency questionnaire
HDL-C: High-density lipoprotein cholesterol
HR: Hazard ratio
ICD: International Classification of Diseases
LDL-C: Low-density lipoprotein cholesterol
MET: Metabolic equivalent
SDS: Self-Rating Depression Scale
TC: Total cholesterol
TCLSIH: Tianjin Chronic Low-grade Systemic Inflammation and Health
TG: Triglycerides

## References

[1] World Health Organization. Depression fact Sheet. 2021.

[2] Cuijpers P, Smit F (2002). Excess mortality in depression: a meta-analysis of community studies. J Affect Disord.

[3] Chisholm D, Sanderson K, Ayuso-Mateos JL, Saxena S (2004). Reducing the global burden of depression: population-level analysis of intervention cost-effectiveness in 14 world regions. Br J Psychiatry.

[4] Yu B, Zhu Q, Meng G, Gu Y, Zhang Q, Liu L, Wu H, Xia Y, Bao X, Shi H (2018). Habitual yoghurt consumption and depressive symptoms in a general population study of 19,596 adults. Eur J Nutr.

[5] Wu H, Zhang S, Meng G, Zhang Q, Liu L, Wu H, Gu Y, Wang Y, Zhang T, Wang X, et al. The consumption of wholegrain is related to depressive symptoms among Chinese adults: a cross-sectional study. Eur J Clin Nutr. 2022;76(1):126–33. 10.1038/s41430-021-00917-2 33931771

[6] Wang J, Um P, Dickerman BA, Liu J (2018). Zinc, magnesium, selenium and depression: a review of the evidence, potential mechanisms and implications. Nutrients.

[7] Hu FB (2002). Dietary pattern analysis: a new direction in nutritional epidemiology. Curr Opin Lipidol.

[8] Nanri A, Kimura Y, Matsushita Y, Ohta M, Sato M, Mishima N, Sasaki S, Mizoue T (2010). Dietary patterns and depressive symptoms among Japanese men and women. Eur J Clin Nutr.

[9] Jacka FN, Pasco JA, Mykletun A, Williams LJ, Hodge AM, O'Reilly SL, Nicholson GC, Kotowicz MA, Berk M (2010). Association of Western and traditional diets with depression and anxiety in women. Am J Psychiatry.

[10] Samieri C, Jutand MA, Feart C, Capuron L, Letenneur L, Barberger-Gateau P (2008). Dietary patterns derived by hybrid clustering method in older people: association with cognition, mood, and self-rated health. J Am Diet Assoc.

[11] Rashidkhani B, Pourghassem Gargari B, Ranjbar F, Zareiy S, Kargarnovin Z (2013). Dietary patterns and anthropometric indices among Iranian women with major depressive disorder. Psychiatry Res.

[12] Xia Y, Wang N, Yu B, Zhang Q, Liu L, Meng G, Wu H, Du H, Shi H, Guo X (2017). Dietary patterns are associated with depressive symptoms among Chinese adults: a case-control study with propensity score matching. Eur J Nutr.

[13] Akbaraly TN, Brunner EJ, Ferrie JE, Marmot MG, Kivimaki M, Singh-Manoux A (2009). Dietary pattern and depressive symptoms in middle age. Br J Psychiatry.

[14] Yin W, Lof M, Chen R, Hultman CM, Fang F, Sandin S (2021). Mediterranean diet and depression: a population-based cohort study. Int J Behav Nutr Phys Act.

[15] Sanchez-Villegas A, Henriquez-Sanchez P, Ruiz-Canela M, Lahortiga F, Molero P, Toledo E, Martinez-Gonzalez MA (2015). A longitudinal analysis of diet quality scores and the risk of incident depression in the SUN Project. BMC Med.

[16] Chocano-Bedoya PO, O'Reilly EJ, Lucas M, Mirzaei F, Okereke OI, Fung TT, Hu FB, Ascherio A (2013). Prospective study on long-term dietary patterns and incident depression in middle-aged and older women. Am J Clin Nutr.

[17] Stephens JD, Althouse A, Tan A, Melnyk BM (2017). The role of race and gender in nutrition habits and self-efficacy: results from the young adult weight loss study. J Obes.

[18] Liu Q, He H, Yang J, Feng X, Zhao F, Lyu J (2020). Changes in the global burden of depression from 1990 to 2017: findings from the global burden of disease study. J Psychiatr Res.

[19] Sudlow C, Gallacher J, Allen N, Beral V, Burton P, Danesh J, Downey P, Elliott P, Green J, Landray M (2015). UK biobank: an open access resource for identifying the causes of a wide range of complex diseases of middle and old age. PLoS Med.

[20] Zung WW (1965). A Self-Rating Depression Scale. Arch Gen Psychiatry.

[21] Yang YWG, Pan X (2009). China food composition.

[22] Liu B, Young H, Crowe FL, Benson VS, Spencer EA, Key TJ, Appleby PN, Beral V (2011). Development and evaluation of the Oxford WebQ, a low-cost, web-based method for assessment of previous 24 h dietary intakes in large-scale prospective studies. Public Health Nutr.

[23] Piernas C, Perez-Cornago A, Gao M, Young H, Pollard Z, Mulligan A, Lentjes M, Carter J, Bradbury K, Key TJ (2021). Describing a new food group classification system for UK biobank: analysis of food groups and sources of macro- and micronutrients in 208,200 participants. Eur J Nutr.

[24] Craig CL, Marshall AL, Sjostrom M, Bauman AE, Booth ML, Ainsworth BE, Pratt M, Ekelund U, Yngve A, Sallis JF (2003). International physical activity questionnaire: 12-country reliability and validity. Med Sci Sports Exerc.

[25] American Diabetes A (2013). Standards of medical care in diabetes–2013. Diabetes Care.

[26] Chobanian AV, Bakris GL, Black HR, Cushman WC, Green LA, Izzo JL, Jones DW, Materson BJ, Oparil S, Wright JT (2003). The seventh report of the joint national committee on prevention, detection, evaluation, and treatment of high blood pressure: the JNC 7 report. JAMA.

[27] Townsend P, Phillimore P, Beattie A (1988). Health and deprivation: Inequality and the North.

[28] Khosravi M, Sotoudeh G, Majdzadeh R, Nejati S, Darabi S, Raisi F, Esmaillzadeh A, Sorayani M (2015). Healthy and unhealthy dietary patterns are related to depression: a case-control study. Psychiatry Investig.

[29] Kim TH, Choi JY, Lee HH, Park Y (2015). Associations between dietary pattern and depression in Korean adolescent girls. J Pediatr Adolesc Gynecol.

[30] Pei Z, Zhang J, Qin W, Hu F, Zhao Y, Zhang X, Cong X, Liu C, Xu L. Association between dietary patterns and depression in chinese older adults: a longitudinal study based on CLHLS. Nutrients 2022, 14(24):5230. 10.3390/nu14245230 PMC9782901 36558386

[31] Le Port A, Gueguen A, Kesse-Guyot E, Melchior M, Lemogne C, Nabi H, Goldberg M, Zins M, Czernichow S (2012). Association between dietary patterns and depressive symptoms over time: a 10-year follow-up study of the GAZEL cohort. PLoS One.

[32] Li Y, Lv MR, Wei YJ, Sun L, Zhang JX, Zhang HG, Li B (2017). Dietary patterns and depression risk: A meta-analysis. Psychiatry Res.

[33] Costa Louzada ML, Martins AP, Canella DS, Baraldi LG, Levy RB, Claro RM, Moubarac JC, Cannon G, Monteiro CA (2015). Ultra-processed foods and the nutritional dietary profile in Brazil. Rev Saude Publica.

[34] Mondal AC, Fatima M (2019). Direct and indirect evidences of BDNF and NGF as key modulators in depression: role of antidepressants treatment. Int J Neurosci.

[35] Molteni R, Barnard RJ, Ying Z, Roberts CK, Gomez-Pinilla F (2002). A high-fat, refined sugar diet reduces hippocampal brain-derived neurotrophic factor, neuronal plasticity, and learning. Neuroscience.

[36] Lopez-Garcia E, Schulze MB, Fung TT, Meigs JB, Rifai N, Manson JE, Hu FB (2004). Major dietary patterns are related to plasma concentrations of markers of inflammation and endothelial dysfunction. Am J Clin Nutr.

[37] Kiecolt-Glaser JK, Derry HM, Fagundes CP (2015). Inflammation: depression fans the flames and feasts on the heat. Am J Psychiatry.

[38] He Y, Li Y, Yang X, Hemler EC, Fang Y, Zhao L, Zhang J, Yang Z, Wang Z, He L (2019). The dietary transition and its association with cardiometabolic mortality among Chinese adults, 1982–2012: a cross-sectional population-based study. Lancet Diabetes Endocrinol.

[39] Rahe C, Unrath M, Berger K (2014). Dietary patterns and the risk of depression in adults: a systematic review of observational studies. Eur J Nutr.

[40] Holscher HD (2017). Dietary fiber and prebiotics and the gastrointestinal microbiota. Gut Microbes.

[41] Yano JM, Yu K, Donaldson GP, Shastri GG, Ann P, Ma L, Nagler CR, Ismagilov RF, Mazmanian SK, Hsiao EY (2015). Indigenous bacteria from the gut microbiota regulate host serotonin biosynthesis. Cell.

[42] Martin CR, Osadchiy V, Kalani A, Mayer EA (2018). The Brain-Gut-Microbiome axis. Cell Mol Gastroenterol Hepatol.

[43] Sarandol A, Sarandol E, Eker SS, Erdinc S, Vatansever E, Kirli S (2007). Major depressive disorder is accompanied with oxidative stress: short-term antidepressant treatment does not alter oxidative-antioxidative systems. Hum Psychopharmacol.

[44] Wang DD, Li Y, Bhupathiraju SN, Rosner BA, Sun Q, Giovannucci EL, Rimm EB, Manson JE, Willett WC, Stampfer MJ (2021). Fruit and vegetable intake and mortality: results from 2 prospective cohort studies of US men and women and a meta-analysis of 26 cohort studies. Circulation.

[45] Nucci D, Fatigoni C, Amerio A, Odone A, Gianfredi V. Red and processed meat consumption and risk of depression: a systematic review and meta-analysis. Int J Environ Res Public Health 2020, 17(18):6686. 10.3390/ijerph17186686 PMC7559491 32937855

[46] Kazemi S, Keshteli AH, Saneei P, Afshar H, Esmaillzadeh A, Adibi P (2021). Red and white meat intake in relation to mental disorders in Iranian adults. Front Nutr.

[47] Zhang S, Gu Y, Bian S, Gorska MJ, Zhang Q, Liu L, Meng G, Yao Z, Wu H, Wang Y (2021). Dietary patterns and risk of non-alcoholic fatty liver disease in adults: a prospective cohort study. Clin Nutr.

[48] Poslusna K, Ruprich J, de Vries JH, Jakubikova M (2009). van't Veer P: Misreporting of energy and micronutrient intake estimated by food records and 24 hour recalls, control and adjustment methods in practice. Br J Nutr.

