# Supplementary material for: Relationship between dietary pattern and depressive symptoms: an international multicohort study
Source: Int J Behav Nutr Phys Act. 2023 Jun 20;20:74. doi: 10.1186/s12966-023-01461-x (PMC10283197; doi:10.1186/s12966-023-01461-x)
Supplement: Supplementary file 1 — Additional file 1. [file 12966_2023_1461_MOESM1_ESM.docx]

| **Supplementary Table 1.** Factor loadings for food items derived from factor analysis (principal component analysis) in the UK Biobank Cohort Study ^a^ | | | | | |  |  |  |  |
| --- | --- | --- | --- | --- | --- | --- | --- | --- | --- |
| **Processed food dietary pattern** | | **Healthy dietary pattern** | | **Meat dietary pattern** | |  |  |  |  |
| **Food groups** | **Factor loadings** | **Food groups** | **Factor loadings** | **Food groups** | **Factor loadings** |  |  |  |  |
| Fat & spreads | 0.70 | Fruits | 0.70 | Meat & meat products | 0.73 |  |  |  |  |
| Cereals & cereal products | 0.67 | Vegetables & potatoes | 0.67 | Alcoholic beverages | 0.58 |  |  |  |  |
| Sugar, preserves, cakes & confectionery, snacks | 0.59 | Dairy & dairy-free products | 0.44 | Egg & egg dishes | 0.27 |  |  |  |  |
| Non-alcoholic beverages | 0.56 | Fish & fish dishes | 0.40 | Vegetables & potatoes | 0.21 |  |  |  |  |
| Mixed-dishes | 0.26 | Nuts & seeds | 0.39 | Non-alcoholic beverages | 0.16 |  |  |  |  |
| Meat & meat products | 0.23 | Non-alcoholic beverages | 0.37 | Sugar, preserves, cakes & confectionery, snacks | 0.14 |  |  |  |  |
| Meat substitutes | 0.19 | Egg & egg dishes | 0.26 | Nuts & seeds | 0.11 |  |  |  |  |
| Dairy & dairy-free products | 0.18 | Cereals & cereal products | 0.11 | Fat & spreads | 0.09 |  |  |  |  |
| Fruits | 0.13 | Meat substitutes | 0.10 | Mixed-dishes | -0.06 |  |  |  |  |
| Vegetables & potatoes | 0.12 | Mixed-dishes | 0.09 | Fish & fish dishes | -0.09 |  |  |  |  |
| Egg & egg dishes | 0.06 | Meat & meat products | 0.07 | Cereals & cereal products | -0.09 |  |  |  |  |
| Alcoholic beverages | 0.04 | Sugar, preserves, cakes & confectionery, snacks | 0.02 | Dairy & dairy-free products | -0.16 |  |  |  |  |
| Nuts & seeds | 0.01 | Alcoholic beverages | -0.04 | Fruits | -0.16 |  |  |  |  |
| Fish & fish dishes | -0.05 | Fat & spreads | -0.15 | Meat substitutes | -0.47 |  |  |  |  |
| **Variance explained (%)** | 13.1 | **Variance explained (%)** | 12.1 | **Variance explained (%)** | 9.6 |  |  |  |  |
| ^a^ participants who completed at least one web-based 24-h dietary assessment in the UK Biobank cohort | | | | | |  |  |  |  |

| **Supplementary Table 2**. Association between dietary patterns and risk of depression symptom in the UK Biobank Cohort Study | | | | | |
| --- | --- | --- | --- | --- | --- |
|  | Quartiles of dietary pattern scores | | | | *P* for trend ^a^ |
|  | Q1 | Q2 | Q3 | Q4 |  |
| **Processed food dietary pattern** |  |  |  |  |  |
| No. of depressive symptom | 349 | 289 | 310 | 355 |  |
| Person years | 177,189 | 177,755 | 177,974 | 177,013 |  |
| Incidence per 1000 person years | 1.97 | 1.63 | 1.74 | 2.01 |  |
| Model 1 | 1.00 (reference) | 1.05 (0.94, 1.18) ^b^ | 1.23 (1.10, 1.38) | 1.37 (1.22, 1.53) | <0.0001 |
| Model 2 | 1.00 (reference) | 1.08 (0.97, 1.21) | 1.26 (1.13, 1.41) | 1.36 (1.20, 1.54) | <0.0001 |
| Model 3 | 1.00 (reference) | 1.08 (0.97, 1.21) | 1.26 (1.12, 1.41) | 1.35 (1.19, 1.53) | <0.0001 |
| **Healthy dietary pattern** |  |  |  |  |  |
| No. of depressive symptom | 295 | 301.00 | 361.00 | 346.00 |  |
| Person years | 177,848 | 177906.00 | 177636.00 | 176541.00 |  |
| Incidence per 1000 person years | 1.66 | 1.69 | 2.03 | 1.96 |  |
| Model 1 | 1.00 (reference) | 0.81 (0.73, 0.91) | 0.76 (0.68, 0.85) | 0.87 (0.79, 0.97) | <0.01 |
| Model 2 | 1.00 (reference) | 0.87 (0.78, 0.97) | 0.83 (0.74, 0.93) | 0.92 (0.83, 1.03) | 0.09 |
| Model 3 | 1.00 (reference) | 0.87 (0.78, 0.97) | 0.83 (0.74, 0.92) | 0.92 (0.82, 1.02) | 0.08 |
| **Meat dietary pattern** |  |  |  |  |  |
| No. of depressive symptom | 336 | 335.00 | 324.00 | 308.00 |  |
| Person years | 177,286 | 177802.00 | 177632.00 | 177211.00 |  |
| Incidence per 1000 person years | 1.90 | 1.88 | 1.82 | 1.74 |  |
| Model 1 | 1.00 (reference) | 0.97 (0.88, 1.08) | 0.91 (0.82, 1.02) | 0.97 (0.86, 1.08) | 0.32 |
| Model 2 | 1.00 (reference) | 1.00 (0.90, 1.11) | 0.93 (0.83, 1.03) | 0.92 (0.82, 1.03) | 0.08 |
| Model 3 | 1.00 (reference) | 1.00 (0.90, 1.11) | 0.93 (0.83, 1.03) | 0.91 (0.81, 1.03) | 0.06 |
| ^a^ Obtained by using multivariable Cox regression model. | | | | | |
| ^b^ Hazard ratios (95% confidence interval) (all such values). | | | | | |
| Model 1 was adjusted for age, sex, and body mass index. | | | | | |
| Model 2 was additionally adjusted for smoking status, alcohol drinking status, married (only in TCLSIH cohort), education level, occupation (only in TCLSIH cohort), visiting friends, living alone, household income (only in TCLSIH cohort), physical activity, total energy intake, Townson depressive index (only in UK Biobank). | | | | | |
| Model 3 was additionally adjusted forfamily history of disease (including cardiovascular disease, hypertension, hyperlipidemia (only in TCLSIH cohort), and diabetes), hypertension, hyperlipidemia (only in TCLSIH cohort), diabetes. | | | | | |
